# Glutathione S-Transferase (GST) Activities and Gene Expression Patterns of Different *GST* Classes in *Musca domestica* L. Depending on Sex and Stage of Development

**DOI:** 10.3390/ijms262311366

**Published:** 2025-11-24

**Authors:** Vladislava Garbaly, Kseniya Krestonoshina, Anna Kinareikina, Svetlana Bobreshova, Anastasiya Avdeeva, Juliya Ismagilova, Ivan Zaitsev, Elena Silivanova

**Affiliations:** All-Russian Scientific Research Institute of Veterinary Entomology and Arachnology—Branch of Federal State Institution Federal Research Centre Tyumen Scientific Centre of Siberian Branch of the Russian Academy of Sciences (ASRIVEA—Branch of Tyumen Scientific Centre SB RAS), Institutskaya St. 2, Tyumen 625041, Russia; gvlada6@mail.ru (V.G.); krutko.k.s@hotmail.com (K.K.); kinareickina@yandex.ru (A.K.); kolyvanova93@mail.ru (S.B.); this_world2000@mail.ru (A.A.); juliya_ismag@mail.ru (J.I.); i.jz@mail.ru (I.Z.)

**Keywords:** insecticide resistance, detoxification enzyme, gene expression, house flies, sex, stage of development

## Abstract

Insecticide resistance in insects poses a serious problem in population control of arthropod vectors and spreaders of human and animal diseases. Metabolic resistance to insecticides is facilitated by detoxification system enzymes, including glutathione-S-transferases (GSTs) involved in phase II of xenobiotic biotransformation. The aim of this study was to analyze the glutathione-S-transferase activity and the expression level of different class *GST* genes in *Musca domestica*. The test subjects were larvae and 3–5-day-old adults of a laboratory susceptible strain (LabTY) and a field deltamethrin-tolerant population (Nik). Based on the LC50 values, the Nik strain showed sensitivity to chlorpyrifos and chlorfenapyr and tolerance to deltamethrin with a remarkable increase in the level of resistance in males compared to females. Expression analysis of eight *GST* genes revealed that the expression of the *GST-E12* gene (Epsilon class) was significantly elevated and the *GST-S1* gene (Sigma class) was significantly decreased in the Nik strain across all groups (larvae, females, and males), with the most pronounced difference in females. A pronounced sexual dimorphism was observed: the expression of most *GST* genes was significantly higher in males than in females in both strains. For the first time, a consistent male-specific overexpression of multiple *GST* genes has been demonstrated in *M. domestica*.

## 1. Introduction

Glutathione-S-transferases (GSTs) (EC 2.5.1.18) are a diverse family of enzymes [1] in which three subfamilies of isoforms are identified: cytosolic, mitochondrial and microsomal [2]. The cytosolic GST form is a superfamily of multifunctional dimeric isoenzymes, which are found in almost all organisms, from microbes to higher animals [2,3,4,5]. In the nucleophilic substitution reaction, GST catalyzes the conjugation of a reduced glutathione (GSH) by its thiol group to various electrophilic chemical substrates [3,6,7]. The mechanism of catalytic action of GST is to reduce the pK of the sulfhydryl group of GSH and convert it into the thiolate (GS−) anionic state at neutral pH, which accelerates its conjugation with substrates [8]. The glutathione conjugates formed are generally less reactive and therefore less toxic [3], as well as more soluble than the parent compounds, which facilitates their exocytic release [2,3,4,5].

Arthropod cytosolic GSTs are mainly involved in adaptation to xenobiotics [2], i.e., they are phase II detoxification enzymes [6]. GST substrates can be both endogenous natural compounds (peroxides, organic hydroperoxides, activated alkenes) and electrophilic xenobiotics [2], such as pesticides [3]. Insecticides can be metabolized either by reductive dehydrochlorination, promoted by GST, or by conjugation reactions with GSH. Additionally, GSTs help to eliminate dangerous types of oxygen-free radicals generated by insecticides [4], providing protection against oxidative stress [2].

Insects possess many different GST isoenzymes [2,3]. For example, about 30 *GST* genes from different subfamilies have been identified in mosquitoes [6]. It is also hypothesized that additional isoenzymes may occur through posttranslational modifications [3]. A mammalian GST nomenclature system has been adopted for insect enzymes, which categorizes them based on amino acid sequences [5] into six classes: delta, epsilon, theta, omega, sigma, and zeta [2,9,10]. The delta and epsilon classes are unique to insects [2,5,11].

The delta and epsilon classes are thought to be involved in neutralizing toxins in insects [1,10]. *Epsilon-GST* overexpression is often associated with insecticide resistance in insects [4,12,13,14,15]. For example, a single treatment with insecticides (chlorpyrifos [12], xanthotoxin [13], permethrin, DDT (1,1,1-trichloro-2,2-bis(4-chlorophenyl)ethane)) [14], chlorantraniliprole [15]) promotes increased expression of *epsilon-GST* isoforms in insect species such as *Cnaphalocrocis medinalis* (Lepidoptera: Crambidae) [12], *Spodoptera litura* (Lepidoptera: Noctuidae) [13], *Bombyx mori* (Lepidoptera: Bombycidae) [14], *Pieris rapae* (Lepidoptera: Pieridae) [15]. In *Aedes aegypti* (Diptera: Culicidae), partial suppression of *GSTe7* and *GSTe2* genes increased insect sensitivity to the pyrethroid deltamethrin that suggests the epsilon-class *GST* genes’ contribution to insecticide resistance [16]. A number of studies have examined the involvement of delta-class *GSTs* in insecticide resistance [17,18]. A notable example of delta-class *GST* involvement is the enzyme *agGSTd1-6* from the malaria mosquito *Anopheles gambiae* (Diptera: Culicidae) [17]. Its elevated activity is directly associated with resistance to DDT, and structural analysis has revealed a specialized hydrophobic active site capable of accommodating the insecticide’s metabolites. Furthermore, in the codling moth *Cydia pomonella* (Lepidoptera: Tortricidae), resistance to the pyrethroid lambda-cyhalothrin is linked to the overexpression of *CpGSTd1* and *CpGSTd3*, with the recombinant *CpGSTd3* protein demonstrating a high capacity to bind this insecticide, implicating the delta class in resistance mechanisms [18]. Additionally, increased expression of 9 out of 15 delta-class *GST* genes was found in the malathion- and propoxur-resistant population of psocids *Liposcelis entomophila* (Enderlein) (Psocoptera: Liposcelididae) from China and several of these genes responded with different expression patterns to malathion, propoxur or deltamethrin exposure [19]. The sigma-class GST is also important for insects. For example, it was shown that the sigma-class GST identified in the honeybee *Apis cerana cerana* (AcGSTS1) was significantly activated in response to various abiotic stresses (cold, heat, UV, H_2_O_2_, HgCl_2_, and insecticides), but with different levels of induction. These results indicated that AccGSTS1 is a critical antioxidant enzyme involved in cellular antioxidant defense and honeybee survival [20].

The house fly *Musca domestica* L. (Diptera: Muscidae) is a global pest of public health and agriculture [21,22,23]. This synanthropic insect is a transmitter for over 100 different diseases in animals and humans, including typhoid fever, dysentery, diphtheria, leprosy, tuberculosis, cholera, anthrax and intestinal parasites [21,23,24]. *M. domestica* L. reproduces at an extremely high rate and develops rapidly [21,25]. The population of this insect species is controlled by humans mainly through insecticides [22,25]. However, the house fly, like other insect species, is able to form resistance to different classes of insecticides [26] (organophosphates, organohalogenides [3], pyrethroids [6], carbamates, organochlorine compounds [4], etc.), which jeopardizes the effectiveness of chemical control strategies [22]. One of the mechanisms for tolerance to insecticides is the enhancement of their metabolism by changing the activity and expression level of certain isoforms of detoxification enzymes [4], including glutathione-S-transferases [2,5]. However, since not all isoenzymes of the GST superfamily are associated with insecticide resistance, it is often difficult to identify specific isoforms involved in this process in *M. domestica* [3]. Two cDNA *MdGST* genes, *MdGST-6A* and *-6B*, which are involved in the development of insecticide resistance [3], were isolated in the ultra-resistant Cornell-HR strain of the house fly. In addition, Sue et al. [27] found that *GST-6A* plays a central role in the development of resistance to organophosphates of the O-alkylphosphate type in organophosphate-resistant strain Yachiyo *M. domestica* L. However, the main role of *GST-6B* that the authors noted was the activation of prothiofos and its increased toxicity in the same house fly strain. The house fly has been shown to possess several delta-class *GST* genes (*MdGST1*–*MdGST4*) [28]. Previously, Fournier demonstrated that *MdGST1* detoxifies organophosphorus insecticides [29].

In this study, the total GST activity and expression levels of different *GST* classes were assessed in house flies of a field population. While several studies have examined *GST* expression in females, data on males remain limited. However, existing sex-specific analyses suggest that males exhibit a wide range of responses to insecticide stress, from high susceptibility to high tolerance, even within the same population. This variation may be attributed to the polygenic nature of resistance and its potential for sex-linked inheritance [30,31]. Consequently, males may serve as a “reservoir” of resistance to insecticides or new xenobiotics, which can be activated under selection pressure. Studies that exclude males are thus biologically incomplete, as they may lead to an underestimation of resistant alleles in the population. The present study evaluated the GST activity in larvae and adults, both females and males, of a field *M. domestica* population in comparison to a laboratory strain. The field population was collected from a livestock farm with a history of pyrethroid use for fly control, prompting an assessment of adults’ sensitivity to deltamethrin and potential cross-resistance to chlorpyrifos and chlorfenapyr. The expression levels of different *GST* classes were determined by focusing on genes encoding GST isoforms with the highest potential contribution to insecticide resistance in the house fly, due to their practical relevance for resistance monitoring. Comparative analysis of the expression levels of these selected genes among larvae, females, and males of the field and laboratory strains identified a potential marker of adaptation to regular insecticide exposure that was independent of developmental stage and sex. This may be useful to specify the possible role of GSTs in the development of insecticide resistance in the house fly, as well as in other species of Diptera insects of economic, medical, and veterinary importance.

## 2. Results

### 2.1. No-Choice Feeding Bioassay

For the correct interpretation of the enzymatic activity and *GST* gene expression results, a toxicological characterization of *M. domestica* strains (Nik, a field population, and Lab TY, a laboratory susceptible strain) with different insecticide sensitivity phenotypes was performed. According to the lethal concentrations for 50% mortality (LC50) and the resistance ratio (RR) values (Table 1), the *M. domestica* adults of the Nik strain were tolerant to the pyrethroid deltamethrin and showed no resistance to the organophosphate chlorpyrifos and the pyrrole chlorfenapyr. It is noteworthy that the LC50 values for most insecticides in males were almost half those for females, with the exception of deltamethrin for the Nik individuals. For the Nik strain, based on the RR, males (RR = 6.73) were more resistant to deltamethrin than females (RR = 2.75).

### 2.2. Glutathione-S-Transferase Activities

**Figure 1:**
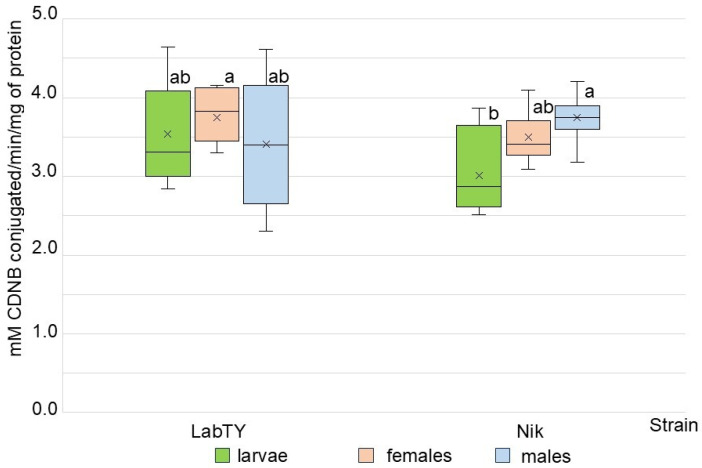
The glutathione-S-transferase activities in *Musca domestica* of the susceptible strain LabTY and the field population Nik. The mean values of the groups with different letters are statistically significant different according to Tukey test (*p* ≤ 0.05).

To identify the biochemical mechanisms underlying the observed toxicological differences, glutathione S-transferase (GST) activity was analyzed. The GST activity values did not differ depending on sex in the LabTY and Nik strains (Figure 1). In the Nik population, the mean value of the GST activity in larvae was 1.24-times statistically significantly lower than that of the males.

### 2.3. Gene Expression Levels of GST Isoforms

To assess which specific isoforms could influence the activity of GST enzymes, and to what extent these differences are due to the origin of the strain and the stage of development, the expression of several *GST* genes was analyzed. Given the limited transcriptomic and functional data available for *M. domestica GSTs*, a comparative genomic approach with the well-annotated and extensively studied model organism *Drosophila melanogaster* provides a valuable strategy for the identification and initial characterization of putative resistance-associated GST isoforms. Genomic prediction in *M. domestica* yielded 33 *glutathione S-transferase (GST)* genes alongside three splice variants. This predicted *GST* repertoire is of a similar scale to that of *D. melanogaster*, which possesses 36 genes and 11 splice variants [32]. Phylogenetic assessment of the cytosolic *GSTs* from both species indicated that the *M. domestica* genes are integrated within the canonical class structure—comprising epsilon, omega, theta, sigma, and zeta—observed in *D. melanogaster*. In addition to the cytosolic forms, four microsomal *GST* genes were identified in *M. domestica*, closely resembling the complement of three genes (producing four isoforms) found in *D. melanogaster* [24]. Based on data from Scott et al. (2014) [24], a table of orthologs of the *GST* genes in *D. melanogaster* and *M. domestica* was compiled (Table 2). To further prioritize candidates with a high potential for involvement in insecticide resistance, we analyzed public RNA-Seq datasets from insecticide-exposed Diptera (including *D. melanogaster*, *Culex pipiens*, and *M. domestica*). The eight *GSTs* were chosen because they represent key classes and are orthologs of genes associated with the resistance in other Diptera, making them the strongest candidates for a focused investigation into metabolic resistance in *M. domestica* populations.

**Figure 2:**
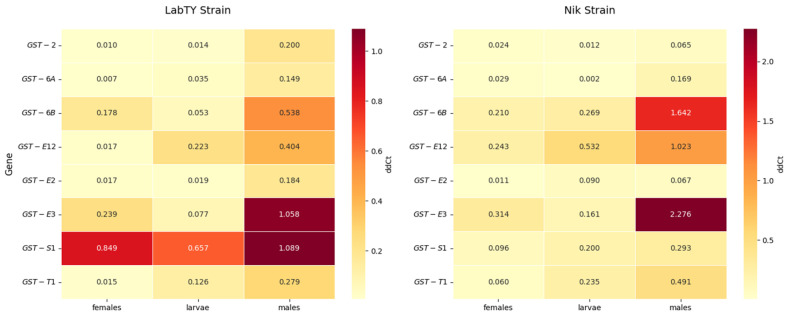
Heat maps of *GST* gene expression in the LabTY and Nik strains *M. domestica*. The maps show the average expression level (ddCt) values from three biological replicates of PCR analysis in different groups (larvae, males, females). Colors represent absolute ddCt values (red = high expression, yellow = low expression). Numbers show mean values from three replicates.

Expression analysis of eight *GST* genes in larvae, males, and females of two strains of the housefly *M. domestica* (LabTY and Nik) revealed significant differences dependent on developmental stage, sex, and genetic background. Statistically significant differences between groups (larvae, males, females) were observed for 7 out of 8 genes (87.5%), while between-strain differences (LabTY vs. Nik) were significant for only two genes (25%) (Figure 2).

Between-strain comparisons revealed significant differences for *GST-E12* (*p* = 0.017) and *GST-S1* (*p* = 0.0004). Expression of *GST-E12* in larvae, males and females of the Nik strain was 2.4-, 2.5- and 14.5- times, respectively, higher compared to the LabTY strain. For *GST-S1*, on the contrary, the expression level in larvae, males and females of the Nik strain was 3.3- (*p* = 0.076), 3.7, and 8.9- times lower, respectively, than in the LabTY individuals. For the remaining genes, the differences did not reach significance (*p* > 0.05), although a trend was observed for *GST-6B* (*p* = 0.0934). Detailed analyses with the Kruskal–Wallis and the nonparametric Dunn’s test within strains showed that in LabTY larvae, *GST-S1* expression was 47.4 times higher than *GST-2* (*p* = 0.023). In Nik larvae, *GST-E12* expression was 222.1 times higher than *GST-6A* (*p* = 0.008). In Nik males, *GST-E3* expression was 35.2 times higher than *GST-2* (*p* = 0.042). In Nik females, *GST-E3* expression was 28.6 times higher than *GST-E2* (*p* = 0.042). In LabTY females, the *GST-S1* expression was 84.9- and 121.3-times higher than that of *GST-2* (*p* = 0.028) and *GST-6A* (*p* = 0.034), respectively.

After pairwise comparison of the expression levels of certain genes between all insect groups, the following statistically significant differences were noted: *GST-6B* expression was 31.2 times higher in Nik males compared to LabTY larvae (*p* = 0.009); *GST-S1* and *GST-E2* were 11.4- (*p* = 0.009) and 16.7-times (*p* = 0.009), respectively, higher in LabTY males compared to Nik females; *GST-E3* was 29.7 times higher in Nik males compared to LabTY larvae (*p* = 0.009); *GST-E12* and *GST-T1* was 60.7- and 33.8-times, respectively, higher in Nik males compared to LabTY females (respectively, *p* = 0.009 and *p* = 0.011).

**Figure 3:**
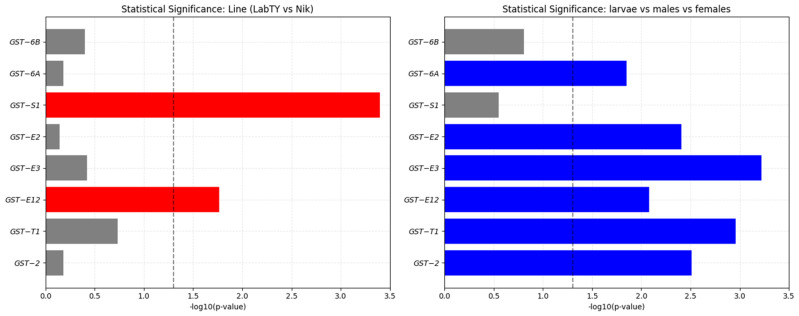
Statistical significance of differences in *GST* gene expression depending on strain and sex/developmental stage *M. domestica*. Red bars indicate genes with significant differences between strains; blue bars indicate genes with significant differences between sexes/developmental stages; gray bars indicate genes without significant differences between strains or sexes/developmental stages. The dotted line represents a significance level of *p* = 0.05. Bars above this line represent statistically significant differences.

In the present study, gene expression of *GST-E12* and *GST-S1* isoforms showed statistically significant differences between strains: *GST-E12* expression was elevated in Nik strain across all stages (up to 14.5-fold in females), while *GST-S1* was significantly higher in LabTY strain (up to 8.9-fold in females). Moreover, *GST-S1* exhibits strain-specific expression patterns without sexual dimorphism, with significantly higher expression in the susceptible LabTY strain compared to the resistant Nik strain, whereas *GST-E12* expression depends on both strain and sex factors (Figure 3). However, no consistent inter-strain expression pattern was observed for the remaining six *GST* genes analyzed. The obtained results suggest that in *M. domestica*, specific *GST* isoforms exhibit strain-specific regulation patterns, which may reflect differential adaptation mechanisms between laboratory and field populations.

## 3. Discussion

This study was conducted to evaluate the GST activity and the expression level of eight *GST* genes in two different strains of the house fly: the susceptible laboratory-derived LabTY strain and the field deltamethrin-tolerant Nik population. The obtained results were compared, firstly, in terms of differences between two populations (field populations versus laboratory susceptible), secondly, for differences in the expression level of *GST*-class genes, and thirdly, the presence of sex-dependent differences in the GST activity and the expression level of genes in insects of both strains was analyzed.

It is known that the level of the GST activity in pyrethroid-resistant insects may not vary [33,34] or could be elevated compared to sensitive specimens [34,35,36]. Kristensen (2005) found a 1.58-fold increase in CDNB-GST activity in females of a field population of house fly resistant to organophosphates and pyrethroids (791a), compared to females of a susceptible strain (WHO) [35]. Elevated levels of GST activity (2.9–5.2 times) were also detected in females of extensively pyrethroid-resistant populations of *M. domestica* from livestock farms in Iran relative to control specimens [36]. Interestingly, after a single exposure to the pyrethroid lambda-cyhalothrin, El Sherif et al. (2022) found no significant changes in GST activity in *M. domestica* larvae of the field population compared to sensitive specimens [25]. These observations are also consistent with the results of other authors who studied the dynamics of resistance development to pyrethroids in *M. domestica*. In the studies by Wang et al. (2012) [37] and Sokolyanskaya (2014) [38] after long-term selection (up to the G30 generation) with pyrethroids (deltamethrin, fenvalerate), higher GST activity in selected specimens of the house fly was observed. And vice versa, a decrease in GST activity in larvae of pyrethroid-resistant field population of *M. domestica* was observed when the exposure to pyrethroids ceased for 25 generations, and as a result, the insect tolerance decreased [39]. In this study, the GST activity of *M. domestica* males of the deltamethrin-tolerant Nik population not differed compared to the susceptible strain (Figure 1). It is noteworthy that females and males of the field population did not differ in GST activity, despite the fact that the Nik males (RR = 6.73) were more resistant to deltamethrin than females (RR = 2.75) of this strain. As mentioned in the review by Enayati et al., 2005 [1], the role of GSTs in insect defense against pyrethroids may involve not only direct catalysis of insecticides via conjugation, but also the sequestration of insecticide molecules and the manifestation of peroxidase activity to neutralize the effects of oxidative stress caused by pyrethroids. However, catalytic activity towards CDNB may not differ between pyrethroid-resistant and sensitive insects. Recent experimental confirmation of the involvement of GSTs in lambda-cyhalothrin resistance via sequestration was provided for *C. pomonella* [18].

Previous studies by other authors have already demonstrated the increased expression of epsilon class *GST* isoforms in pyrethroid-resistant insect populations [16,40,41,42,43]. The studies were mainly focused on insect vectors of medical and veterinary importance—mosquitoes of genera *Culex*, *Aedes* and *Anopheles*. In *Aedes aegypti*, *Anopheles funestus*, and *Anopheles sinensis* (Diptera: Culicidae) mosquitoes resistant to DDT and the pyrethroids (permethrin, deltamethrin) researchers have observed increased expression of *GSTe2*, *GSTe3*, *GSTe4*, *GSTe5*, and *GSTe6* genes [16,40,41,43]. For other pyrethroid resistant insect species, such as *Rhynchophorus ferrugineus* (Coleoptera: Curculionidae), high expression of *GSTd1-4* and *GSTe1-8* has been reported [42]. In the study by Chen et al. (2023) on a field population of *Diaphorina citri* (Hemiptera: Liviidae) resistant to the pyrethroid fenpropathrin, most of the *GST* isoforms in adult insects without sex separation did not change transcript levels or there was even a decrease in transcript levels [44].

Analysis of *GST* gene expression revealed pronounced sexual dimorphism across both strains studied. Male-specific overexpression was observed for multiple *GST* genes: *GST2* expression was 20.5-fold higher in LabTY males compared to LabTY females, while *GST-E12* showed 4.2-fold higher expression in Nik males than in Nik females. Similar male-biased patterns were detected for *GST-E3*, *GST-E2*, *GST-6A*, and *GST-6B*, indicating a consistent trend across different *GST* classes. The represented data suggest a predominant influence of sexual dimorphism and developmental stage on *GST* gene expression, with peak levels in males, which may be related to their enhanced role in xenobiotic detoxification in the gonads or during mating. This sexual dimorphism in *GST* expression aligns with previous findings in other insect species. Studies in *Ae. aegypti* reported higher expression of epsilon class *GSTs* in males of pesticide-resistant populations [16]. Similarly, research on *D. melanogaster* demonstrated significantly higher GST activity in males compared to females under both control and pesticide-exposure conditions [45]. These observations suggest that sexual dimorphism in detoxification enzyme expression may be a widespread phenomenon in insects. The biological significance of male-biased *GST* expression in *M. domestica* requires further investigation. While the specific functional implications for insecticide resistance remain to be fully elucidated, the consistent overexpression of multiple *GST* genes in males across both laboratory and wild strains suggests potential differences in detoxification capacity between sexes. This pattern may reflect underlying physiological or ecological factors influencing GST regulation in male and female houseflies.

The pronounced male-biased expression of multiple *GST* genes observed in the present study represents a contrasting pattern to previously reported female-specific detoxification mechanisms in *M. domestica*. The probability of sex differences in mechanisms of insecticide resistance in *M. domestica* was previously demonstrated using spinosad as an example [46,47,48]. For instance, the study results of *CYP6A1*, *CYP6D1* and *CYP6D3* expression levels in males and females of spinosad-, fipronil- and imidacloprid-resistant strains of *M. domestica* indicated that the involvement of cytochromes P450 in the development of resistance to spinosad is more specific for females than for males [46]. In another study, the expression levels of *CYP4G2*, *CYP6A5v2* and *MdαE7* genes were statistically significantly higher in spinosad-resistant *M. domestica* females than in susceptible females, while the expression levels of these genes were lower in spinosad-resistant males than in susceptible males [47,48]. This divergence suggests that sexual dimorphism in insecticide resistance mechanisms may be compound-specific and involve different detoxification enzyme families. The coexistence of both male-biased (GST) and female-biased (P450) resistance mechanisms within the same species highlights the complexity of metabolic adaptation in *M. domestica* and suggests potential sex-specific evolutionary trajectories in response to different insecticide selection pressures.

## 4. Materials and Methods

### 4.1. Insects

The research was carried out with individuals of two *Musca domestica* strains, namely the LabTY and the Nik. The LabTY is the laboratory susceptible strain obtained in 2009 from Novosibirsk Agrarian University (Russian Federation). The Nik is the field population that was captured in May–September 2023 in livestock houses of the Tyumen Region (Russian Federation), where pyrethroid deltamethrin-based formulations were used against houseflies for several seasons. The 18–19th generation of the Nik population was used in this study. The Lab TY and the Nik strains are kept in insectariums of the ASRIVEA—Branch of Tyumen Scientific Centre SB RAS without contact to insecticides.

The larvae of *M. domestica* were kept in 800 cm^3^ glass beakers and reared on an artificial diet consisting of 55 g of bran and 150 mL of water until the pupation stage. Following eclosion, adult flies were kept in nylon cages with metal frames measuring 25 cm × 25 cm × 25 cm. They were provided with a diet of dry baby food products and water, and were maintained under standard conditions at 24–28 °C, 60–80% relative humidity and a 12 h/12 h (light/dark) photoperiod.

### 4.2. No-Choice Feeding Bioassay

The no-choice feeding bioassay was used for laboratory tests of the chlorpyrifos technical substance (technical substance, 97%, Jiangsu Inter-China Group Corporation, Zhenjiang, China), deltamethrin (Delcid, 4%, AVZ S-P) and chlorfenapyr (Pyrafen EC, 360 g/L, AgroServer.ru, Moscow, Russia) toxicity against adults of *M. domestica*. Flies starved for 12 h prior to the tests. Acetone solutions of insecticides (30 µL) were used to soak the sugar (0.1 g) in glass cups, and in the control test, the sugar was treated with pure acetone in the same volume. After the acetone evaporated, 10 flies of each sex (separated by sex) of 3–5 days old were placed in each cup. The cups were sealed with mesh pistons from the top and supplied with water drinkers. The mortality of the flies was recorded after 48 h for chlorpyrifos and deltamethrin and 72 h for chlorfenapyr. Insecticides were tested at 6–8 concentrations that led to insect mortality from 0% to 100%. Each concentration was tested at least three times and the tests were carried out on different days.

### 4.3. Glutathione-S-Transferase Activity

Homogenates were prepared from 45 insect abdomens of each strain, which were pooled into 15 separate samples both for females and males. From each larva, at least 15 individual homogenate samples were prepared for both the LabTY and the Nik strain. Homogenates were prepared from insects on a Bioprep-24 homogenizer (Hangzhou All-sheng Instruments Co., Ltd., Hangzhou, China) at +4 °C with the addition of 0.1 M phosphate buffer pH 7.6, 1 mM EDTA (ethylenediaminetetraacetic acid), 1 mM PTU (N-phenylthiourea), 1 mM PMSF (phenylmethylsulfonyl fluoride), 1 mM DTE (1,4-dithioerythritol). The supernatant obtained after centrifugation (7000× *g*, 2 min, 4 °C) was used to determine the enzymatic activity of GST [49] and the quantitative protein content by the Lowry protein assay with using bovine serum albumin for the calibration curve. For the LabTY strain, the protein content in homogenates was (mean ± standard deviation) 4.14 ± 0.74 mg/mL, 3.82 ± 0.33 mg/mL and 4.44 ± 0.60 mg/mL for larvae, females and males, respectively. For the Nik strain, the protein content in homogenates was (mean ± standard deviation) 3.81 ± 0.41 mg/mL, 3.21 ± 0.36 mg/mL and 3.35 ± 0.57 mg/mL for larvae, females and males, respectively. Activity towards 1-chloro-2,4-dinitrobenzene (CDNB) was monitored at 340 nm in the kinetic mode for 20 min at intervals of 1 min intervals at 25 °C. The reaction mixture contained 15 µL of homogenate and 195 µL of 100 mM potassium phosphate buffer (pH = 6.5) with 9 mM of GSH in 1 mM of CDNB. To account for the non-enzymatic conjugation, 15 µL of water was added to the reaction mixture instead of a homogenate. Specific enzyme activity was calculated considering non-enzymatic substrate conversion, absorption coefficient (9.6 mM^−1^ cm^−1^), assay volume, homogenate volume, dilution factor, path length, and protein content of the sample, and it was expressed as mM CDNB conjugated in 1 min per mg of protein (mM CDNB conjugated/min/mg protein). Two analytical replicates were performed for each homogenate sample.

### 4.4. Gene Expression Levels of GST Isoforms

The selection of candidate *glutathione S-transferase* (*GST*) genes for this study was based on a comprehensive analysis of public transcriptomic data and established orthology. To identify *GST* genes with a high potential for involvement in insecticide detoxification, relevant RNA-Seq datasets from the NCBI SRA database were analyzed. The present research specifically focused on experiments involving the exposure of Diptera species to various insecticides, including: *D. melanogaster* treated with chlorfenapyr (SRA accessions: SRR16043570-SRR16043572), *Culex pipiens* treated with deltamethrin (SRA accessions: SRR1645076, SRR1645095), *M. domestica* treated with spinosad (SRA accessions: SRR1802194-201). Differential expression analysis of these datasets allowed us to compile a list of *GST* genes that were consistently and significantly upregulated in response to insecticide challenge across these different species (Table 3). This list was then integrated with orthology data from a published whole-genome study of *M. domestica* [24].

Larvae and the abdominal section of adult insects (at least 15 males and 15 females of each strain) were sampled to investigate gene expression levels. Total RNA was isolated using RiZol (diaGene, Moscow, Russia), additionally treated with DNAase I. The quantity and quality of total RNA were evaluated spectrophotometrically on a Nano-300 device (Allsheng, Hangzhou, China) according to the ratio of the optical density at 260/280 nm wavelength (absorbance ratio 1.9–2.1). The integrity of the total RNA fractions was verified by using 1% agarose gel electrophoresis. The first strand of cDNA was synthesized using the MMLV RT kit (Evrogen, Moscow, Russia) according to the manufacturer’s instructions. Primers were developed using Primer3Plus 3.3.0 software. Real-time PCR was performed using 5X qPCRmix-HS SYBR mix (Evrogen, Moscow, Russia) on a Gentier 96E amplifier (Tianlong, Xi’an, China). EF-1 and RP49 genes were used as reference genes [50,51]. Each reaction was repeated at least three times; non-templated and negative controls were used to detect reagent contamination.

The primer specificity testing for RT-qPCR was performed in several steps: primer sequence alignment in BLAST NCBI 2.17.0; PCR product melting-curve analysis (Table S1); electrophoresis of PCR product in 1.5% agarose gel (Figure S1). Except for the annealing temperature, the amplification conditions were identical for each gene: initial denaturation at 95 °C (5 min), denaturation—40 cycles at 94 °C (1 min), annealing according to primer temperature (20 s), elongation at 72 °C (20 s), and final elongation at 72 °C (5 min) (Table 3).

### 4.5. Statistical Analysis

The dose–response mortality in no-choice feeding bioassays was analyzed by probit regression analysis to calculate lethal concentrations for 50% (LC50) and 95% (LC95) mortality for 95% confidence interval (CI) [52]. The resistance ratio (RR) was calculated as the ratio of LC50 of the insecticide for flies of the Nik strain to LC50 for Lab TY flies. Susceptibility of the field population to the insecticides was determined based on the following criteria: RR < 1—high susceptibility to the insecticide, RR = 1–2—absence of resistance (the population is sensitive to the insecticide), RR = 3–10—very low resistance (the insects are tolerant to the insecticide), RR = 11–30—medium resistance (the insects are moderately resistant), R = 31–100—high resistance (the insects are resistant to the insecticide), RR > 100—very high resistance (the insects are highly resistant to the insecticide). Relative gene expression data (ddCt values) were analyzed using Python (version 3.9) with the pandas, matplotlib, seaborn, and scikit-learn libraries. Pivot tables were generated by aggregating mean ddCt values for each *GST* gene across developmental stages and sexes (larvae, females, males) separately for the LabTY and Nik strains, followed by visualization as heat maps. Statistical analysis of the results was performed using StatSoft Academic Analyst 2.5 and Statistica Academic 13 to calculate descriptive statistics (means, standard deviations) and to assess the statistical significance of differences. For the GST enzymatic activity results, one-way analysis of variance (ANOVA) with Tukey test for multiple comparisons was applied. Gene expression results were analyzed using the Kruskal–Wallis test followed by pairwise comparison using the nonparametric rank test (Dann’s test for analysis within a strain or within a single gene, Mann–Whitney test for analysis between strains) that is appropriate for small samples and does not assume normal data distribution. All *p*-values were corrected for multiple comparisons where applicable, with a significance level of α = 0.05.

## 5. Conclusions

The present study provides a comprehensive analysis of the glutathione S-transferase (GST) system in the house fly, *Musca domestica*, comparing a susceptible laboratory strain (LabTY) with a field population (Nik). Toxicological profiling confirmed that the Nik population exhibits sensitivity to chlorpyrifos and chlorfenapyr and tolerance to deltamethrin (RR = 2.75–6.73) with a remarkable increase in the level of resistance in males despite the absence of differences in enzyme activity by sex in adults. Among eight genes studied, only *GST-E12* (Epsilon class) showed a consistent and significant upregulation in the Nik strain across all developmental stages and sexes, making it a promising candidate biomarker for pyrethroid resistance in *M. domestica*. In contrast, *GST-S1* (Sigma class) was significantly more expressed in the LabTY strain, suggesting its role may be more related to basal metabolic functions than to insecticide-specific resistance.

To conclude, the integrated analysis reveals that pyrethroid tolerance in the house fly is characterized by the specific upregulation of key isoforms like *GST-E12*. Crucially, the detoxification landscape is profoundly influenced by sex, with males exhibiting a consistently heightened expression of multiple *GST* genes. For a more complete and in-depth understanding of the specific mechanisms of resistance to pyrethroids in female and male house flies and the contribution of different classes of *GSTs*, additional studies are needed on the level of expression and activity of GSTs in insecticide-treated individuals. This research provides not only specific molecular targets for resistance monitoring but also fundamentally advances the understanding of the complex, sex-biased nature of metabolic adaptation in *M. domestica*.

## Figures and Tables

**Table 1:** Toxicity of chlorpyrifos, deltamethrin, and chlorfenapyr to *Musca domestica* adults of the laboratory strain (Lab TY) and the field strain (Nik, G18-19).

Insecticide	Strain	N	LC50 (95% CI) ^a^	LC95 (95% CI) ^a^	Slope (±SE)	χ^2^	RR (95% CI)
Chlorpyrifos	LabTY ♀	195	12.227 (8.055–18.561)	84.627 (55.748–128.465)	2.19 (±0.09)	0.21	-
	LabTY ♂	195	7.592 (5.479–10.520)	25.043 (18.073–34.702	3.21 (±0.07)	0.32	-
	Nik ♀	195	14.962 (11.497–19.472)	32.653 (25.091–42.494)	4.89 (±0.06)	0.52	1.22 (1.05–1.43)
	Nik ♂	195	6.363 (4.794–8.445) ^#^	15.659 (11.798–20.782)	4.27 (±0.06)	0.85	0.84 (0.80–0.88)
Deltamethrin	LabTY ♀	240	2.078 (1.295–3.334)	26.880 (16.755–43.123)	1.65 (±0.11)	0.17	-
	LabTY ♂	233	1.004 (0.688–1.465)	5.240 (3.591–7.647)	2.40 (±0.08)	0.61	-
	Nik ♀	195	5.707 (3.811–8.545) *	24.012 (16.037–35.953)	2.64 (±0.09)	0.88	2.75 (2.56–2.95)
	Nik ♂	195	6.730 (4.036–11.224) *	56.806 (34.065–94.728)	1.78 (±0.11)	0.98	6.73 (5.85–7.68)
Chlorfenapyr	LabTY ♀	384	23.625 (17.385–32.103)	96.306 (70.872–130.868)	2.72 (±0.06)	0.05	-
	LabTY ♂	401	9.745 (7.320–12.975) ^#^	58.781 (44.149–78.262)	1.64 (±0.10)	0.72	-
	Nik ♀	230	27.609 (19.906–38.293)	139.574 (100.632–193.584)	2.44 (±0.07)	0.55	1.17 (1.15–1.19)
	Nik ♂	230	14.332 (10.526–19.513) ^#^	55.686 (40.900–75.817)	2.84 (±0.07)	0.87	1.47 (1.44–1.50)

^a^ μg of active substance per one g of sugar; N—the number of adult flies used in bioassay including control; resistance ratio (RR) = LC50 of the Nik strain/LC50 of the Lab TY strain; ♀—females; ♂—males; * statistically significant differences compared to the LabTY strain based on non-overlapping confidence intervals (*p* ≤ 0.05); ^#^ statistically significant differences compared to the females of the same strain based on non-overlapping confidence intervals (*p* ≤ 0.05).

**Table 2:** Orthologues of the *GST* genes in *D. melanogaster* and *M. domestica*.

*GST* Class	*D. melanogaster*	*M. domestica*			
		Protein Accession	Gene ID	mRNA Accession	Gene Name
Epsilon	*DMGSTE4-8*	XP005185166	LOC101895036	XM_005185109.4	*GST-2*
		XP005185169	LOC101895555	XM_005185112.4	*GST-6B*
		XP005190027	LOC101887250	XM_005189970.4	*GST-1*
		NP_001295979.2	-	NM_001309050.2	*GST-6A*
	*DMGSTE13A-B*	XP005179508	LOC101900016	XM_005179451.4	*GST-1*
		XP005179509	LOC109611606	XM_005179452.4	*GST-1*
	*DMGSTE11*	XP005184662	LOC101895316	XM_005184605.4 (X1)	*GST-E11*
		XP005184663		XM_005184606.4 (X2)	
		XP005184664	LOC101895607	XM_005184607.4 (X1)	*GST-1*
	*DMGSTE14*	XP005180753	LOC101888181	XM_005180696.4	*GST1-like*
		XP005180754	LOC101888349	XM_005180697.4	*GST1-like*
Delta	*DMGSTD11A-B*	XP005180103	LOC101897797	XM_005180046.4 (X1)	*GST-D11*
	*DMGSTD10*, *1A-B*	XP005180099	LOC101897094	XM_005180042.4 (X1)	*GST4-like*
		NP_001295926.1	LOC101897277	NM_001308997.1	*GST-2*
Theta	*DMGSTT4*	XP005191555	LOC101897781	XM_005191498.4	*GST-T1*
	*DMGSTT3A-B*	XP005177600	LOC101900949	XM_005177543.4	*GST-T3*
Sigma	*DMGSTS1*	NP_001273827.1	-	NM_001286898.1	*GST*

**Table 3:** Genes evaluated in the present study.

Gene Name	GeneID	Nucleotide Sequence (5′→3′) of Primers (Forward/Reverse)	Ta, °	Length of PCR Product (bp)	GenBank ID
*RP49* *	LOC101894827	GTTATGCCAAATTGTCGCACA GGCGGGTACGTTTGTTGG	59.5	123	XM_020038490.2
*EF-1*	LOC101899175	TAAGGAAGGTAACGCTGAAGG CAAGGGCAAACGCAAAGG	59.5	91	XM_005181459.4
*GST-6A*	101887423	ATTCGACGACAAAATGGG CCTTAGCCATCAAATTAACC	61.5	131	NM_001309050.2
*GST-6B*	LOC131804118	ACCTGTTCGTGCTTGTTTGC CGAGTGTGGGCACTGTATGT	63.5	137	XM_059126509.1
*GST-S1* (*GST*)	101890455	TGGAAGTTAACGGCAAGCGT CCGGCAAAGTAGACATCGGC	61.5	313	NM_001286898.1
*GST-T1*	LOC101897781	GTGTAGCCATATTCCGCCACT GCATCGTACCATTTTGAGAGCT	59.5	391	XM_005191498.4
*GST-E2* (*GST-2*)	LOC101895036	AAGCGATCACAACAGCCAAC CATCGTCTTCCAGTGTAGGCA	61.5	240	XM_005185109.4
*GST-E3* (*GST-6B*)	LOC101895555	ATCCCCAACATACAGTGCCC TCCCTGAAATAAGACACCAGCT	61.5	182	XM_005185112.4
*GST-2*	LOC101897277	AGAACGGACAACAAGTAGCTCC AATGGCACGAGATTCCCACA	59.5	247	NM_001308997.1
*GST-E12*	LOC101900672	GGTCACTTGTTTGCCCGTCT GCACCCTCCTCGTTTGTATCC	61.5	302	XM_059131016.1

* also known as *RPL32* gene.

## Data Availability

The raw data supporting the conclusions of this article will be made available by the authors on request.

## Supplementary Materials

The following supporting information can be downloaded at: https://www.mdpi.com/article/10.3390/ijms262311366/s1.

## Abbreviations

The following abbreviations are used in this manuscript:

GSTs	Glutathione-S-transferases
LC50	Lethal concentrations for 50% mortality
LC95	Lethal concentrations for 95% mortality
RR	Resistance ratio
CI	Confidence intervals
WHO	World Health Organization
ddCt	Relative quantity of gene expression
