# Supplementary figures and images for: Glutathione S-Transferase (GST) Activities and Gene Expression Patterns of Different GST Classes in Musca domestica L. Depending on Sex and Stage of Development
Source: Int J Mol Sci. 2025 Nov 24;26(23):11366. doi: 10.3390/ijms262311366 (PMC12692679; doi:10.3390/ijms262311366)

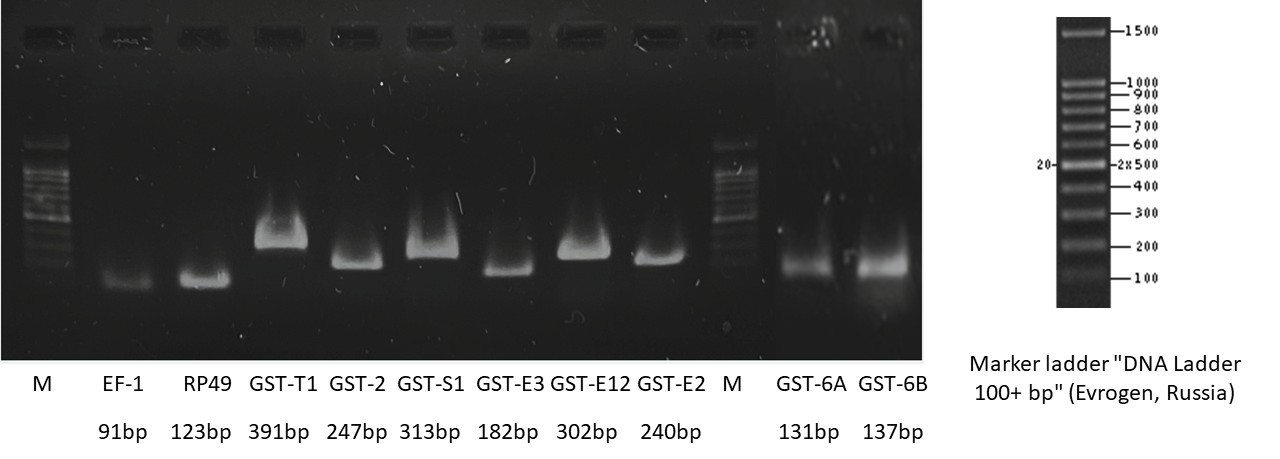

Supplement: Supplementary file 1 [file ijms-26-11366-s001.zip › Figure S1 (Electrophoresis of 8 GST genes + 2 reference genes used in the study; M - marker ladder).jpeg]
